# Physical activity and the rejuvenation of Connswater (PARC study): protocol for a natural experiment investigating the impact of urban regeneration on public health

**DOI:** 10.1186/1471-2458-13-774

**Published:** 2013-08-23

**Authors:** Mark A Tully, Ruth F Hunter, Helen McAneney, Margaret E Cupples, Michael Donnelly, Geraint Ellis, George Hutchinson, Lindsay Prior, Michael Stevenson, Frank Kee

**Affiliations:** 1 UKCRC Centre of Excellence for Public Health, Queen’s University Belfast, Belfast, Northern Ireland; 2 Centre for Public Health, Queen’s University Belfast, Belfast, Northern Ireland; 3 Institute of Child Care Research, Queen’s University Belfast, Belfast, Northern Ireland; 4 Department of General Practice, Queen’s University Belfast, Belfast, Northern Ireland; 5 School of Planning Architecture and Civil Engineering, Queen’s University Belfast, Belfast, Northern Ireland; 6 School of Biological Sciences, Queen’s University Belfast, Belfast, Northern Ireland; 7 School of Sociology, Social Policy and Social Work, Queen’s University Belfast, Belfast, Northern Ireland

**Keywords:** Physical activity, Built environment, Natural experiment, Mixed methods, Urban regeneration, Behaviour change, Sustainability, Cost-effectiveness, Walkability

## Abstract

**Background:**

There is a dearth of evidence regarding the impact of urban regeneration projects on public health, particularly the nature and degree to which urban regeneration impacts upon health-related behaviour change. Natural experiment methodology enables comprehensive large-scale evaluations of such interventions. The Connswater Community Greenway in Belfast is a major urban regeneration project involving the development of a 9 km linear park, including the provision of new cycle paths and walkways. In addition to the environmental improvements, this complex intervention involves a number of programmes to promote physical activity in the regenerated area. The project affords a unique opportunity to investigate the public health impact of urban regeneration.

**Methods/Design:**

The evaluation framework was informed by the socio-ecological model and guided by the RE-AIM Framework. Key components include: (1) a quasi-experimental before-and-after survey of the Greenway population (repeated cross-sectional design), in tandem with data from a parallel Northern Ireland-wide survey for comparison; (2) an assessment of changes in the local built environment and of walkability using geographic information systems; (3) semi-structured interviews with a purposive sample of survey respondents, and a range of community stakeholders, before and after the regeneration project; and (4) a cost-effectiveness analysis. The primary outcome is change in proportion of individuals identified as being regularly physically active, according to the current UK recommendations. The RE-AIM Framework will be used to make an overall assessment of the impact of the Greenway on the physical activity behaviour of local residents.

**Discussion:**

The Connswater Community Greenway provides a significant opportunity to achieve long-term, population level behaviour change. We argue that urban regeneration may be conceptualised meaningfully as a complex intervention comprising multiple components with the potential, individually and interactively, to affect the behaviour of a diverse population. The development and implementation of our comprehensive evaluation framework reflects this complexity and illuminates an approach to the empirical, rigorous evaluation of urban regeneration. More specifically, this study will add to the much needed evidence-base about the impact of urban regeneration on public health as well as having important implications for the development of natural experiment methodology.

## Background

Most people in high income countries lead inactive lives, fuelling the rise in non-communicable diseases [1]. Physical inactivity has been classified as the fourth leading cause of death worldwide [2]. The potential of the built environment to influence population levels of physical activity was recognised by the World Health Organization [3], and subsequently in the UK Foresight report [4]. However much of the evidence on the relationship between factors such as the design of local neighbourhoods and the provision of opportunities for physical activity has come from cross-sectional studies [5]. The UK Foresight report highlighted the need for evidence of the effectiveness of environmental interventions to help to sustain behaviour changes [4].

A review of gaps in the evidence for physical activity promotion highlighted a need for socio-ecological studies of community interventions [6]. A systematic review of walking to promote physical activity further emphasised the need to study the effects of larger scale community interventions [7]. The UK National Institute for Health and Care Excellence (NICE) review of physical activity interventions prioritised research on the evaluation of effectiveness of environmental interventions on physical activity, especially those including a contemporaneous comparison group [5]. Therefore, there is a gap in the evidence investigating the public health impact of community-wide, environmental change interventions.

Given the scale, it is impossible for researchers to implement built environment interventions and therefore the use of natural experiments has often been the only option to evaluate the impacts of such interventions on health and wellbeing. Natural experiments are interventions that are not under the control of researchers, but often employ observational methods to understand the effects, compared to a control or comparison group [8]. The method enables researchers to evaluate the effectiveness of ‘real world’ public health interventions in the absence of randomised controlled trials [9]. The MRC guidelines for natural experiments recommended the specification of *a priori* hypotheses, clear definitions of target populations, explicit sampling criteria and the use of valid and reliable measures of outcomes [8].

### Aim and objectives

The aim of this study is to determine the impact of a systems-wide community intervention to promote physical activity in the context of a major inner city urban regeneration project.

Specific objectives include evaluating:

(i) The impact of a suite of community-based interventions to promote physical activity;

(ii) The role of the local built environment, and of individual, community and organisational networks in sustaining change; and,

(iii) The cost-effectiveness of this socio-ecological and systems-based approach to effecting physical activity behaviour change.

## Methods/Design

### The intervention

The Connswater Community Greenway (CCG) [10] is an example of a natural experiment which afforded an opportunity to evaluate the public health impact of a major urban regeneration project in Belfast, Northern Ireland, funded by a £32 million Big Lottery Living Landmarks Award. There are 29 electoral wards in the political constituency of the Greenway with a total population of approximately 110,600, and 22 wards (approximately 87,500 residents) with a geographical centroid within a 1 mile radius. Seven of the wards are within the top 25% most deprived wards in Northern Ireland, as determined by the 2005 Northern Ireland Multiple Deprivation Measure [11]. The aim of the regeneration project is to offer enhanced opportunities for physical activity and outdoor recreation through specific environmental improvements including the construction of 19.4 km of new cycle and walkways and the provision of allotments. In addition, the regeneration aims to improve the aesthetics of shared public spaces, involving the planting of trees/shrubs, erection of public art and the remediation of water courses to improve the natural diversity and reduce the risk of flooding. It is also planned that 24 hour-a-day lighting, CCTV and the presence of park wardens will result in a changed perception of safety in the community. In addition to the range of environmental improvements, a number of interventions to promote physical activity in the CCG area, ranging across the individual, community and environmental dimensions will be implemented. Examples of these interventions include the extension of neighbourhood walking groups and other physical activity initiatives targeted to promote the use of the Greenway in distinct population segments (e.g. young mothers, unemployed and senior citizens), schools-based initiatives and a variety of community-based social marketing initiatives. Theory-based Intervention Mapping will inform the development of the design of these interventions [12], ensuring that physical activity interventions are tailored to meet the needs of the local community [13].

### Overall research design

The PARC Study is a before-and-after evaluation of the effects of the CCG on physical activity and health, and comprises of four main elements:

1. A quasi-experimental before-and-after survey of the CCG population (repeated cross-sectional design), utilising data from a parallel Northern Ireland-wide survey for comparison;

2. Assessment of change in the local built environment and walkability using geographic information systems (GIS);

3. Semi-structured interviews with 60 residents, and a range of community stakeholders before and after the regeneration project;

4. A cost-effectiveness evaluation.

The study has been approved by the Office for Research Ethics Committees, Northern Ireland (09/NIR02/66).

#### Summative evaluation

##### Survey design

**Figure 1:**
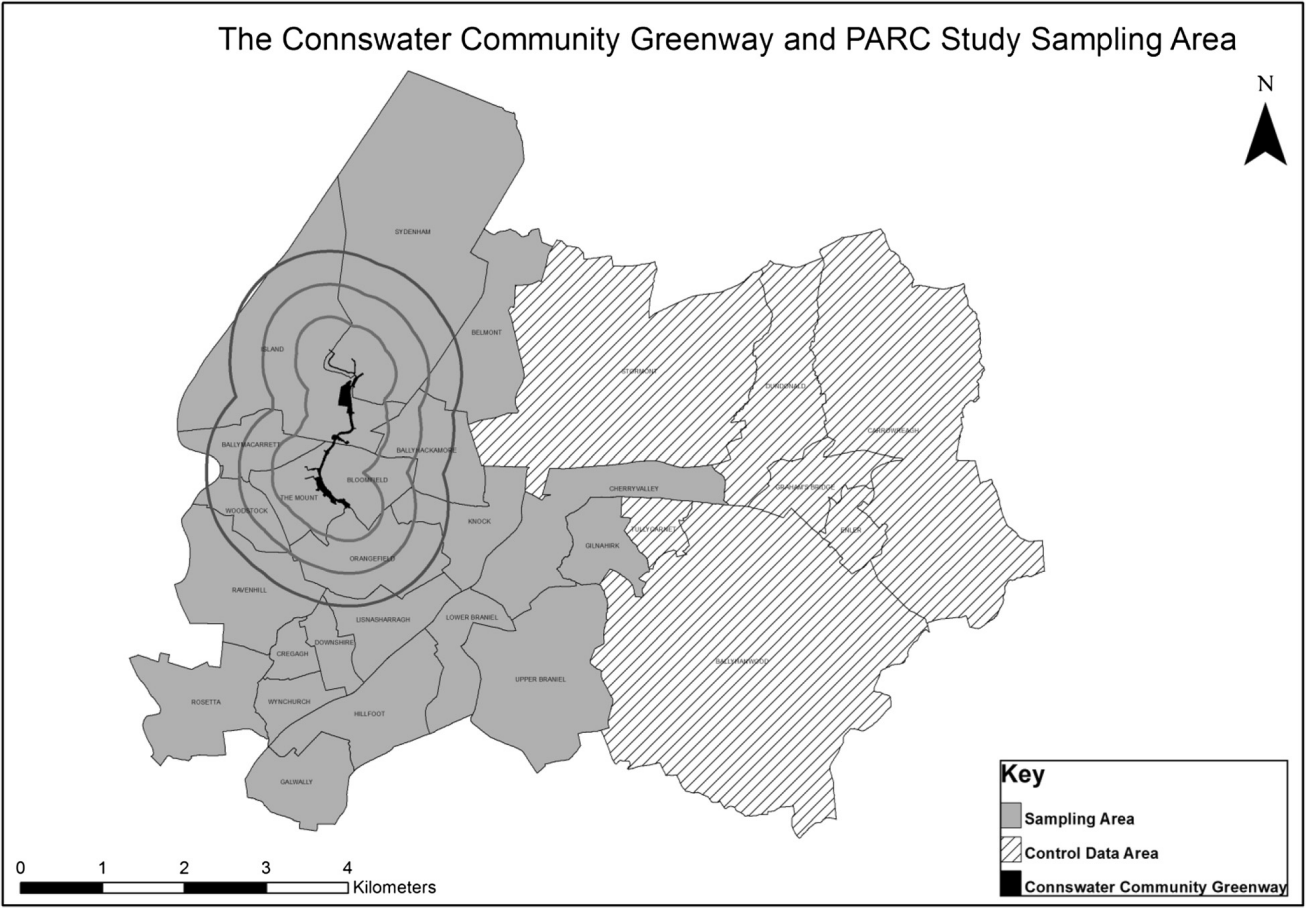
**The Connswater Community Greenway and PARC Study Sampling Area.** PA=physical activity.

A survey of a random selection of households will be conducted before-and-after the regeneration of the CCG. The target area was selected as 29 electoral wards identified during the development of the CCG project (Figure 1). The sampling strategy will ensure proportionality with the Northern Ireland population based on estimates of the number of residents aged 16 or older provided by the 2001 Census. The surveys will each take place over a 12-month period and a random probability sampling framework will be constructed by a random selection of addresses from each of the selected output areas using the Royal Mail’s Postal Address File (PAF), stratified by the proportion of the overall population within each area. An information sheet about the study will be posted to each household, which will be followed up by a visit approximately 1-2 weeks later from a trained interviewer. If no one is present at the time of interview, up to five call-backs will be made in order to achieve a completed interview. If no response to call backs, the address will be recorded and another address within the same area will be selected from the PAF using the same selection process. For each household, the ‘last birthday rule’ (person in the household aged 16 years and over who had the most recent birthday) will be applied to randomly select an individual aged 16 years and over within each selected household to complete the survey. After the individual has given written consent to participate, an interviewer-administered questionnaire will be completed.

##### Survey outcome measures

**Figure 2:**
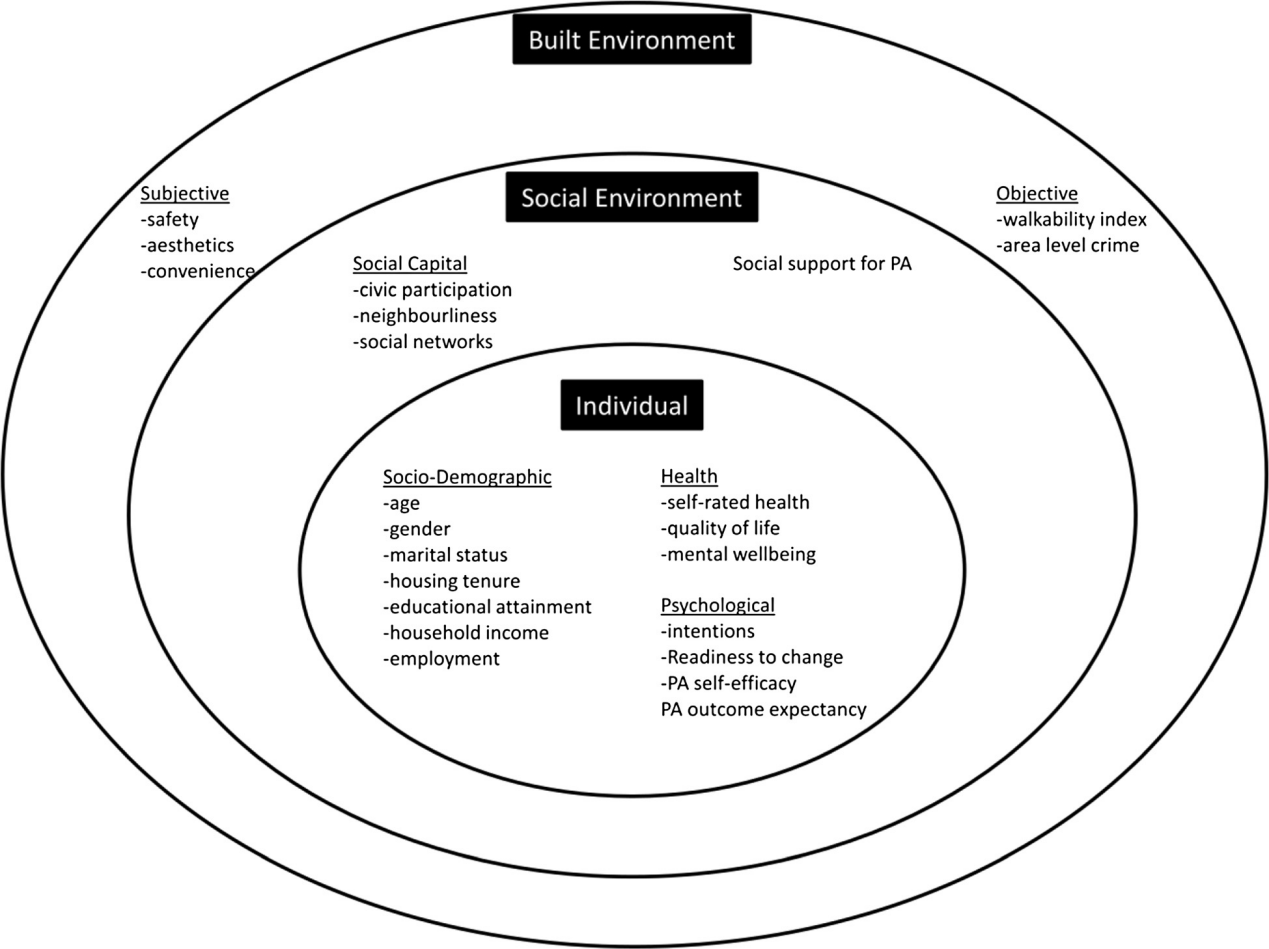
**Overview of the evaluation framework informed by the socio**-**ecological model (adapted from Sallis et al 2012 **[51]**).**

The survey content is informed by, and reflects the various levels of, the socio-ecological model comprising measures of individual, community and environmental factors (Figure 2). The primary outcome will be the change in proportion of individuals identified as regularly physically active, according to the current UK recommendations of a minimum of 150 minutes of moderate-intensity physical activity per week [14]. Physical activity will be assessed using the Global Physical Activity Questionnaire (GPAQ) [15]. GPAQ assesses levels of overall physical activity through the accumulation of occupational, transport, and recreational physical activities of moderate and vigorous intensity, and was developed by the World Health Organisation to allow comparison of physical activity across countries.

Outcomes which may moderate/mediate the relationship between changes in the built environment and physical activity will be assessed. These include (i) perceptions of the characteristics of the environment associated with active travel and physical activity, including aesthetics, safety and walkability [16] - a “walkability index” for each Super Output Area will be created (based on land use characteristics and street connectivity [17,18]) using GIS, to explore the relationship between high and low “walkable” environments and physical activity; (ii) self-rated health using the EQ-5D-3L instrument [19,20]; (iii) health related quality of life using the Short-Form 8 Health Survey (SF-8) [21]; (iv) mental well-being using the Warwick-Edinburgh Mental Well-being Scale [22,23]; (v) common core theoretical constructs such as intention, stage of change, self-efficacy and outcome expectancy based on the outcomes from the 2001 NIH sponsored workshop and studies to develop a unified theory of behaviour change applied to physical activity [24,25]; (vi) items relating to neighbourhood social capital, as reflected in civic engagement, neighbourliness, social networks and support, and perceptions of the local area using the instrument employed in the UK General Household Survey [26,27]; (vii) awareness of the Greenway, assessed using items similar to those employed in an evaluation of the VERB campaign [28] - this will be used to assess the possible mediating effects of social marketing activities to promote physical activity in the CCG area; (viii) individual socio-demographic variables including age, gender, marital status, housing tenure, educational attainment, household income level and employment status. Access to a bicycle and the number of vehicles available for use by the household will also be recorded. In addition, using a tailored questionnaire, a range of discrete choice questions will assess preferences for physical activity with reference to the local built environment, amenities and transport infrastructure [29].

To control for the possible influence of weather patterns on physical activity, data for the seven days preceding interview will be collated from the nearest weather station via the UK MET office. This will include daily rainfall, hours of sunshine, minimum and maximum temperature and wind speed.

##### Nested accelerometer-enhanced sub-study

In addition to participating in the household survey, a randomly selected sub-sample of 100 residents will be invited to wear an Actigraph GT3X accelerometer (Actigraph Inc, FL) at both time points (before and after CCG construction) to provide an objective measure of physical activity for comparison with self-reported questionnaire data (GPAQ). Participants will be asked to wear the accelerometer from the time they wake-up to the time they go to bed, for seven consecutive days. Standard cut-off values for moderate and vigorous physical activity will be used to calculate total minutes of moderate and vigorous physical activity per week [30].

##### Comparison survey

Comparison data will be drawn from Northern Ireland-wide surveys of physical activity in approximately 4,000 randomly selected individuals from the general population at the same two time points [31]. This will provide contemporaneous data to compare any changes in physical activity in the target area with that of the general population, or of similar urban areas in Northern Ireland, outside the study area [31]. These individuals will be sampled and interviewed in a similar manner to the methods described above.

##### Sample size and analyses

The sample size was estimated using the same method as Cochrane et al. [32], based on Cohen’s arcsine transformation [33]. We estimated the initial population proportions of those achieving the recommended levels of physical activity using alternative assumptions of 20%, 30% and 50%. Therefore the sample size required to detect differences in population proportions is 934 at both time points, assuming an effect size of 0.15 at 90% power (α = 0.05). We will survey 950 individuals who reside in the electoral wards whose geographic centroid is within one mile of the CCG area, before and after the intervention. In addition, we will survey a further 289 individuals who reside in other wards in the wider area (representing a similar proportion of the population to the other areas), which will allow us to explore distance decay. Since the “comparator” community survey undertaken by SportNI will involve approximately 4,000 individuals across Northern Ireland, we will essentially have a large control group providing additional power to demonstrate any differences that might emerge in the CCG population.

Area-by-category classifications of change in the proportion of the population meeting the physical activity guidelines will be compared by cross-tabulation and the difference in distributions assessed for statistical significance using the χ^2^ test. Multinomial logistic regression will be used to test a prediction model for categories of change in physical activity by area/age/income bracket, allowing a comparison of odds ratios for the various categories of physical activity by area [34]. A key strength of the analysis will be the capacity to make multiple comparisons between the CCG area and similar groups and areas across Northern Ireland as well as the capacity to analyse trends in physical activity behaviours according to distance between respondents’ homes and the Greenway.

### Process evaluation

In addition to the household survey, a comprehensive process evaluation, informed by the socio-ecological model, will be conducted before and after CCG construction (Figure 2).

#### Individual level

##### Semi-structured interviews

A purposive maximum variation sample of 60 households representing varying distance from the Greenway, and individuals of a range of age, gender and socio-economic position (SEP) will be selected and invited to participate in detailed semi-structured interviews. Participants will be identified from respondents to the household survey. The interviews will explore the effects of key determinants on physical activity behaviour. Specific topics will include (i) perceptions of own health, its determinants and lifestyle behaviours, (ii) perceptions of the barriers and facilitators to healthy lifestyles, (iii) effects of local environment on behaviours, motivation and personal health, (iv) knowledge of the Greenway and related local health promotion initiatives, and (v) perceptions of the quality of the local environment, including safety issues and expectations from the urban regeneration. The same participants will be interviewed at two time points, pre and post CCG construction. The qualitative data emerging from this series of interviews will add richly to the explanatory power of our quantitative data [35].

#### Community level

##### Focus groups

Physical activity intervention design and implementation can benefit from meaningful participation of the community and relevant agencies. Focus groups (n=14) will be conducted with community stakeholders utilising the networks and knowledge of local community agencies. This collaborative approach will facilitate purposive sampling to recruit adults for the focus group discussions representing the diverse population of the study area (e.g. age, gender, SEP). Topics explored will include engagement in physical activity, barriers and facilitators to physical activity in the local area, views on local facilities and opportunities for physical activity and the perceived usefulness of the proposed Greenway to encourage local residents to be more active. Focus groups will be audio-recorded and data will be analysed using a mixture of text-based content analysis and thematic analysis [36,37].

#### Organisational level

##### Network analysis

The sustainability of any efforts to support behaviour change at both individual and organisational levels will depend on the strength and empowerment of the community and inter-organisational networks. An assessment of the processes that facilitate or inhibit the effective implementation of the intervention strategy will form an integral part of the overall design [38]. The evolution of community capacity and the effectiveness of these partnerships to meet their health promotion objectives will be assessed before and after the CCG construction period using established partnership and social network analysis tools and techniques [39,40]. Briefly, the strength and extent of network ties between the statutory and voluntary CCG stakeholders, in terms of reciprocal relationships, trust, exchange of information, technical assistance, referrals or funding, will be evaluated using a range of mathematical parameters describing the network. This includes the network density, degree and between-ness centrality, which reflect respectively the connectedness of the network by the proportion of possible ties in a network that are present, the number of ties to and from a stakeholder (used to determine opinion leaders), and the frequency that one lies between others thus occupying a strategic position on the network [41]. The evolution of these measures over the duration of the CCG construction will be an important dimension of the outcome evaluation. This will be complemented by semi-structured interviews with key CCG stakeholders, including local health professionals, employers, retailers and activists from voluntary and statutory bodies, to evaluate the impact of the Greenway on the effectiveness of the partnership and on knowledge mobilisation. All qualitative proceedings will be audio-recorded and analyzed using a form of thematic analysis [36] whereby the identified themes and sub-themes will be represented in terms of a matrix for further analysis [37].

#### Built environment

##### Geographic information systems (GIS)

In order to examine the specific influence of changes to the built environment, GIS data of the environmental features in the area at the finest available spatial level (varying from address of the individual household to Super Output Area level) pre- and post-regeneration will be collected. This will include mapping of the footpath network (“Real Walkable Network”) across the study area, topography, transport features, physical activity opportunities, for example, green space, local amenities, parks, using a mix of aerial photography, and checked using open source spatial data and aerial photography. The network will be tested and validated by peer checking of mapped elements and ground-truthing (observational checks) to assure its quality. The development and validation of the Real Walkable Network will be the subject of a future publication. These data will be used to revise the “walkability index” for a 500 m and 1000 m buffer zone around the household of each respondent to the household survey. Analysis will include an assessment of the changes in walkability of the neighbourhoods and the influence of distance decay (exposure to the regeneration project) on physical activity behaviour. We will also map and describe individual household level change in objectively measured accessibility to the regenerated, environmental physical activity opportunities and examine the association between change to the built environment and change in physical activity and other health-related outcomes.

##### Usage of the greenway

Direct observation of usage of the Greenway will be conducted in two ways: (i) conducting before-and-after Intercept Surveys of CCG use (at four locations on the Greenway), adapting SUSTRANS methodology; (ii) employing the SOPARC (System for Observing Play and Recreation in Communities) methodology, a validated technique to assess the use being made of green space and parks in the area before and after the construction of the CCG [42]. We will recruit volunteers from the local community, who will undertake a period of training, to help with data collection at eight target areas using the SOPARC protocol, over the course of seven days, both in winter and summer. These data will be complemented by assessing the quality of built environment features using a validated instrument, Environmental Assessment of Public Recreation Spaces (EAPRS) [43]. Analysis will explore the characteristics of Greenway users, the types of activities they are involved in and the relationship between parks/green space use and environmental quality.

Further, effective mechanisms are in place to collect information to assess the reach, ‘dose’ and fidelity of intervention implementation and of the resources used. The Belfast Health and Social Care Trust and the East Belfast Community Development Agency will collect core data on the number of people enrolling and completing various physical activity promotion initiatives in the CCG area.

##### Other data

During the construction of the Greenway, aspects of local or regional transport infrastructure and policy may change, with implications for the active travel behaviour of local residents. Changes will be monitored using routinely available information, including bus timetables and traffic survey data collated by the Department for Regional Development (DRD). The latter will yield comparative information on average annual distances travelled by all forms of transport and access to and use made of public transport. DRD also collects routine information on bicycle use via electronic monitors dispersed throughout the city. In addition, effects on road traffic accidents will be monitored using routinely available statistics, compiled by the Police Service of Northern Ireland (PSNI). The effect of the urban regeneration on tenancy turnover rates, the quality of local social housing stock, and crime statistics in the target area and in neighbouring areas will also be monitored using Northern Ireland Housing Executive and PSNI data respectively.

##### Economic evaluation

Conducting an economic evaluation of environmental interventions that promote physical activity is fraught with methodological difficulties and multiple approaches have been recommended [44]. Firstly, we will use a cost-effectiveness approach and adapt the PREVENT model [45], collecting information about the costs of the Greenway construction and interventions in the CCG area and the outputs in terms of the reduction in the proportion of the population categorised as physically inactive, thereby permitting us to derive Incremental Cost-effectiveness Ratios (ICERs). The impact on ICERs of varying the model assumptions will be explored in subsequent sensitivity analyses. Secondly, an alternative insight will be gained from a behavioural economics perspective in which, using a contingent valuation and choice experiment [46], we will forecast the impact of the built environment, local amenities and infrastructure on physical activity participation and health and derive the impact of these on “Willingness-to-Pay”.

##### RE-AIM Framework

The RE-AIM Framework will be used to make an overall assessment of the impact of the Greenway on the physical activity behaviour of local residents, so that we have a clearer understanding of the **R**each, **E**ffectiveness, **A**doption, **I**mplementation and **M**aintenance of any changes wrought by the regeneration [47,48]. This framework allows concurrent evaluation of dimensions considered relevant to ‘real world’ implementation, such as the capacity to reach socially-disadvantaged populations and the changes in health-related outcomes, such as physical activity. In particular, we will examine differences across social groups and whether the intervention has impacted on inequalities in physical activity participation and health in the local community.

## Discussion

A major urban regeneration project has afforded a unique opportunity to evaluate a ‘real world’ natural experiment. The CCG is a complex intervention with multiple interacting components affecting a diverse population. Many challenges must be overcome in the development and implementation of an evaluation of such an intervention, requiring both methodological innovation and development.

### Challenges in natural experiments

In response to the Foresight report [4] and Wanless report [49], highlighting the weak evidence for public health interventions, a field of research utilising natural experiments for evaluating public health interventions has emerged. Studies of this kind raise a number of scientific and evaluative challenges, for example, aligning research timetables with the regeneration timelines, rapidly recruiting and conducting a baseline assessment prior to implementation of the intervention and, measuring confounders and levels of exposure. Health behaviours, such as physical activity, are complex behaviours that require multifaceted interventions and a composite evaluation framework. Meta-evaluations required for such a complex intervention need to be scientifically robust yet flexible to cope with unpredictable implementation and a changing environment which is not controlled by the researcher.

### The RE-AIM Framework

The RE-AIM Framework has been used to guide the evaluation of interventions that address the different levels of the socio-ecological model, including individual, community, organisational, and population level. King and colleagues [48] have refined the RE-AIM Framework to evaluate the effects of environmental change approaches to enhancing population health. We have further adapted this model to evaluate a complex, systems-wide community intervention involving changes to the built environment. The RE-AIM Framework provides a useful template to guide the design and implementation of a comprehensive public health evaluation of a large built environment intervention, expanding the assessment of interventions beyond efficacy to address multiple criteria that better assess the potential for dissemination and public health impact of health promotion interventions.

### Multi-disciplinarity

A comprehensive evaluation framework has been developed by a multi-disciplinary research team comprising of academics from public health, economics, sociology, psychology, statistics and spatial planning. The research team has actively engaged with the local community partnerships, city council, health practitioners and a variety of government departments in planning the evaluation framework. Previous research suggests that building meaningful partnerships of diverse communities can improve health outcomes [50]. The PARC Study has a strong community engagement ethos and the research team will be involved in transferring knowledge from its research activities to the East Belfast community and beyond, through articles in newssheets and summaries of key research findings on the CCG website. Members of the research team will present findings from the PARC Study quarterly at the Greenway Stakeholders Forum, Politicians’ Breakfast, and the Community Workers Forum. Findings will also be disseminated through peer-reviewed publications and national and international conference presentations.

## Abbreviations

CCG: Connswater Community Greenway; DRD: Department for Regional Development; EAPRS: Environmental Assessment of Public Recreation Spaces; EQ-5D: EuroQol-5D; GIS: Geographic Information Systems; GPAQ: Global Physical Activity Questionnaire; ICER: Incremental Cost-effectiveness Ratio; PAF: Postcode Address File; PARC: Physical Activity and the Rejuvenation of Connswater; PSNI: Police Service of Northern Ireland; RE-AIM: Reach, Effectiveness, Adoption, Implementation, Maintenance; SEP: Socio-economic Position; SF-8: Short Form 8 Health Survey; SOPARC: Systems for Observing Play and Recreation in Communities; SportNI: Sport Northern Ireland.

## Competing interests

The authors declare that they have no competing interests.

## Authors' contributions

All authors contributed to the development of the study protocol and to the critical revision of the paper and approved the final version. FK (Principal Investigator) had the original idea for the study and led the design of the study and the application for grant funding. MT, HMcA, GE, GH, LP, MD, MS, and MC (co-investigators) contributed to the design and writing of the original protocol and funding application. RH and MT wrote the first draft of the manuscript.

## Pre-publication history

The pre-publication history for this paper can be accessed here:

http://www.biomedcentral.com/1471-2458/13/774/prepub
